# Role of connexin channels in the retinal light response of a diurnal rodent

**DOI:** 10.3389/fncel.2014.00249

**Published:** 2014-08-25

**Authors:** Angelina Palacios-Muñoz, Maria J. Escobar, Alex Vielma, Joaquín Araya, Aland Astudillo, Gonzalo Valdivia, Isaac E. García, José Hurtado, Oliver Schmachtenberg, Agustín D. Martínez, Adrian G. Palacios

**Affiliations:** ^1^Facultad de Ciencias, Centro Interdisciplinario de Neurociencia de Valparaíso, Universidad de ValparaísoValparaíso, Chile; ^2^Departamento de Electrónica, Universidad Técnico Federico Santa MaríaValparaíso, Chile; ^3^Instituto de Sistemas Complejos de ValparaísoValparaíso, Chile

**Keywords:** retina, physiology, neural coding, multi-electrode array (MEA), connexins

## Abstract

Several studies have shown that connexin channels play an important role in retinal neural coding in nocturnal rodents. However, the contribution of these channels to signal processing in the retina of diurnal rodents remains unclear. To gain insight into this problem, we studied connexin expression and the contribution of connexin channels to the retinal light response in the diurnal rodent *Octodon degus* (degu) compared to rat, using *in vivo* ERG recording under scotopic and photopic light adaptation. Analysis of the degu genome showed that the common retinal connexins present a high degree of homology to orthologs expressed in other mammals, and expression of Cx36 and Cx43 was confirmed in degu retina. Cx36 localized mainly to the outer and inner plexiform layers (IPLs), while Cx43 was expressed mostly in cells of the retinal pigment epithelium. Under scotopic conditions, the b-wave response amplitude was strongly reduced by 18-β-glycyrrhetinic acid (β-GA) (−45.1% in degu, compared to −52.2% in rat), suggesting that connexins are modulating this response. Remarkably, under photopic adaptation, β-GA increased the ERG b-wave amplitude in degu (+107.2%) while reducing it in rat (−62.3%). Moreover, β-GA diminished the spontaneous action potential firing rate in ganglion cells (GCs) and increased the response latency of ON and OFF GCs. Our results support the notion that connexins exert a fine-tuning control of the retinal light response and have an important role in retinal neural coding.

## Introduction

It is well-accepted that gap junction channels are major components of the nervous system that mediate both electrical and metabolic coupling between neurons and glial cells (Demb and Pugh, 2002; Connors and Long, 2004; Sohl et al., 2005; Bloomfield and Volgyi, 2009). Electrical synapses are formed by gap junctions, which are composed of connexins subunits (Guldenagel et al., 2000; Sohl et al., 2000; Willecke et al., 2002). Many studies of the last decade have revealed the widespread expression of connexins in the nervous system, including the retina (Kar et al., 2012; Saez and Leybaert, 2014) of mammals with different life styles, e.g., primates, guinea pig, ground squirrel, rabbit, rat and mouse. Cx36, which forms channels with small unitary conductance and low voltage sensitivity, is expressed in rodent retina (Al-Ubaidi et al., 2000) in cone and rod photoreceptors, bipolar cells (BCs), AII amacrine cells and ganglion cells (GCs) (Feigenspan et al., 2001, 2004; Guldenagel et al., 2001; Mills et al., 2001; Deans et al., 2002; DeVries et al., 2002; Hidaka et al., 2002, 2004; Lee et al., 2003; Degen et al., 2004; Han and Massey, 2005; Schubert et al., 2005; Dedek et al., 2006; O'Brien et al., 2012). Other connexins are also expressed in retinal neurons. Cx45 has been detected in subpopulations of BCs, amacrine cells and GCs (Lin et al., 2005; Maxeiner et al., 2005; Dedek et al., 2006), and Cx57 in horizontal cells (HCs) (Massey et al., 2003; Hombach et al., 2004; Ciolofan et al., 2007). Moreover, Cx43 is expressed in Müller cells (Janssen-Bienhold et al., 1998; Johansson et al., 1999; Kihara et al., 2006) and cells of the retinal pigment epithelium (Janssen-Bienhold et al., 1998).

The functional contribution of each retinal connexin type has only partly been unveiled. For example, deletion of the Cx36 gene in mouse reduces the b-wave of the electroretinogram (ERG) under scotopic conditions (Guldenagel et al., 2001; Maxeiner et al., 2005), and eliminates ON-center GC responses (Deans et al., 2002). Previous studies have shown that retinal gap junctions are regulated by several factors, including light, circadian rhythm as well as neuromodulators such as nitric oxide and dopamine (Bloomfield and Volgyi, 2009). These factors may induce post-translational modifications of Cxs. For example, under photopic conditions, phosphorylation of mouse Cx36 modulates ON BCs and AII amacrine cell synapses (Kothmann et al., 2009). Limited evidence is available regarding the importance of retinal connexins in nocturnal vs. diurnal species. Lee et al. (2003) have shown that Cx36 is expressed in the retina of guinea pig, a crepuscular rodent. More information has been obtained from studies in zebrafish, a diurnal teleost fish, in which coupling between photoreceptors is controlled by phosphorylation of Cx35, an ortholog of mammalian Cx36, which is part of the mechanisms that control light and dark adaptation cycles (Li et al., 2009). To further understand the role of connexins in the retina of diurnal species, we studied the retinal expression of connexins Cx36 and Cx43 and the general contribution of connexins to retinal light responses in *Octodon degus* (degu), a crepuscular diurnal rodent (Ardiles et al., 2013) that presents a high percentage of cone photoreceptors (30%) (Jacobs et al., 2003) with different spectral sensitivities (500 nm M-cones and 360 nm UV S-cones) (Chavez et al., 2003). The results were compared to rat, a standard nocturnal model with a low percentage of cones (1–3%). We found that general blockage of connexin channels with 18-β-glycyrrhetinic acid (β-GA) (Xia and Nawy, 2003; Pan et al., 2007) *in vivo* and *in vitro* had similar effects on the scotopic light response, but opposing results under photopic adaptation in both rodent species, supporting a differential role of connexins in the retinal cone pathways of diurnal vs. nocturnal species.

## Materials and methods

### Animals

Adult male and female *Octodon degus* and *Sprague dawley* rats were maintained in the animal facility of the Universidad de Valparaiso, at 20–25°C on a 12-h light-dark cycle, with access to food and water *ad-libitum*. All experiments were approved by the bioethics committee of the Universidad de Valparaiso, in accordance with the bioethics regulation of the Chilean Research Council (CONICYT), and had an approved animal welfare assurance (NIH A5823-01).

### Western blots

After ERG experiments, the animals were deeply anesthetized with halothane and decapitated. The right eye (control) was removed immediately and opened along the *ora serrata*. The lens and vitreous humor were removed and the isolated retinas were homogenized in ice-cold lysis buffer containing 50 mM Tris-HCl pH 7.4, 150 mM NaCl, 0.1% Triton X-100, 0.6 mM PMSF and a cocktail of proteases (Sigma P8340) and phosphatase inhibitors (Sigma P5726), at 4°C. The homogenates were centrifuged at 13,000 g for 20 min at 4°C. The supernatant was collected and the total protein contents were determined according to the method of Bradford. Aliquots of tissue samples corresponding to 40 μg of total protein were heated to 100°C for 5 min with Laemmli sample buffer (300 mM Tris-HCl pH 6.8, 50% glycerol, 10% SDS, 500 mM DTT, 0.05% β-mercaptoethanol, 0.01% bromophenol blue) and loaded onto 10% polyacrylamide gels. The proteins were blotted to polyvinyl difluoride membrane (Amersham Biosciences, Buckinghamshire, UK) and blocked for 1 h at room temperature in PBS with 0.2% Tween-20 and 8% non-fat dry milk. The membrane was incubated overnight at 4°C with a mouse monoclonal anti-Cx35/36 antibody (MAB 3045, Chemicon; dilution 1:1000); or a rabbit polyclonal anti-Cx43 antibody (C-6219, Sigma; dilution: 1:300) in blocking solution. The membrane was rinsed in PBS with 0.2% Triton X-100 (four times, 15 min each time) and incubated for 2 h at room temperature with a 1:1000 dilution of horseradish peroxidase-conjugated goat anti-mouse or anti-rabbit IgG antibody (Jackson Immuno Research Laboratories). Thereafter, the blot was washed in PBS with 0.2% Triton X-100 and bound immunoglobulins were visualized with SuperSignal West Pico chemiluminescent substrate (Pierce Biotechnology, Rockford, IL, USA) on Kodak BioMax Light film.

### Immunohistochemistry

For immunohistochemistry, eyes were fixed in 4% paraformaldehyde (PFA) in PBS (0.1 M, pH 7.4) for 10 min. A small hole was cut out with a fine needle through the *ora serrata*, and the eye was immersed again in 4% PFA (2 h at 4°C) followed by three washes in PBS. For cryoprotection, the fixed eye was immersed in sequentially increasing concentrations of sucrose in PBS (10%, 20%, 1 h in each concentration and finally in 30% at 4°C overnight). Thereafter, the eye was frozen in tissue freezing medium (Tissue-Tek OCT Compound, Sakura Finetek Europe). Frozen sections of 15 μm thickness were obtained with a cryostat (Leica CM 1900, Germany) at −20°C, and mounted on poly-L-lysine coated microscope slides. To wash out the cryomatrix, the sections were rinsed four times with PBS plus 0.3% Triton X-100 (TBS). Nonspecific binding was blocked by incubating the sections for 1 h at room temperature in TBS containing 10% normal goat serum (NGS). Retinal sections were incubated overnight at 4°C with the primary antibodies diluted in the blocking solution: Mouse monoclonal anti-Cx35/36 (MAB 3045, Chemicon; dilution 1:200), or rabbit polyclonal anti-Cx43 (C-6212, Sigma; dilution 1:300). The sections were washed four times with TBS for 15 min and incubated for 2 h at room temperature with the secondary antibodies diluted in blocking solution; Cy3-conjugated affinity pure Goat anti-mouse IgG antibody (113-166-072, Jackson Immuno Research Lab; dilution: 1.1000) or Cy2-conjugated affinity pure Goat anti-rabbit IgG antibody (111-226-047, Jackson Immuno Research Lab; dilution 1:1000). Some slices were incubated for 30 min with propidium iodide (dilution 1:8000 in TBS) to stain nuclei. The retinal sections were mounted in Fluomount (Dako Industries, Carpenteria, CA, USA). In control experiments, non-specific binding was tested by omitting the primary antibody. The slices were imaged using a spinning disk confocal microscope Olympus (BX-DSU, Olympus, Japan) and captured using an ORCA-2 camera (Hamamatsu Photonics, Japan). Images were acquired and processed using the Cell-R program (Olympus Soft Imaging Solutions, Germany) and processed using AutoQuantX 2.2.2 (Media Cybernetics, USA) deconvolution software.

### Bioinformatics

**Figure 1:**
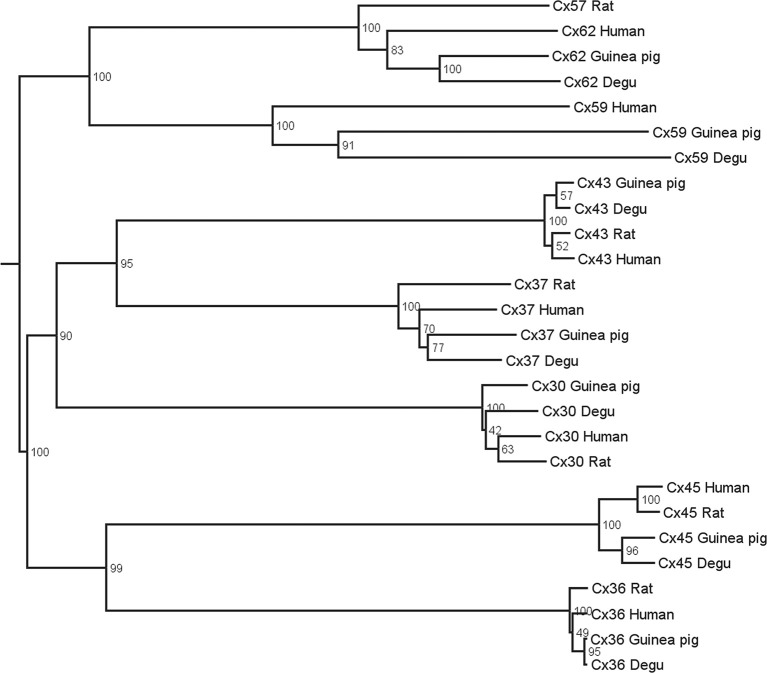
**Phylogenetic tree of gap junctions present in the retina of different mammals**. Multiple alignments with subsequent neighbor-joining and bootstrap analysis were performed on protein sequences of human, guinea pig, degu, and rat. Connexin orthologs tend to group together whereas the paralog sequences are further apart.

Multiple alignments followed by neighbor joining and bootstrap analyses were performed to align protein sequences of different connexins genes from human, guinea pig, degu and rat. Connexins of the α, β, γ, and δ groups were included in the dendrogram. Gap junction orthologs tend to group together whereas paralog sequences are further apart (Volgyi et al., 2013a). All sequences for degu shown in Figure 1 were extracted from PUBMED (Gene). The species and protein sequence IDs used for this analysis are: *Homo Sapiens* Cx43 NP_000156.1, Cx37 NP_002051.2, Cx59 NP_110399.2, Cx62 NP_115991.1, Cx30 NP_001103689.1, Cx45 NP_005488.2, Cx36 NP_065711.1; *Cavia porcellus* Cx43 NP_001166219.1, Cx37 XP_003471533.1, Cx59 XP_005008590.1, Cx62 XP_005008615.1, Cx30 XP_005007068.1, Cx45 XP_003465784.1, Cx36 XP_003475711.1; *Octodon degus* Cx37 XP_004641059.1, Cx59 XP_004648949.1, Cx62 XP_004649057.1, Cx30 XP_004639430.1, Cx45 XP_004633888.1, Cx36 XP_004643177.1, CX43 XP_004630289.1; *Rattus norvegicus* Cx43 NP_036699.1, Cx37 NP_067686.1, Cx57 NP_001166979.1, Cx30 NP_445840.1, Cx45 NP_001078850.1, Cx36 NP_062154.1.

### *In vivo* ERG

All procedures and the optical stimulation apparatus of the ERG system have been described previously (Chavez et al., 2003; Peichl et al., 2005). In brief, the a-wave ERG corresponds to the photoreceptor response and the b-wave to the depolarization of ON BCs (Brown, 1968). For scotopic conditions, animals were pre-adapted for 2 h to total darkness, and for photopic conditions, they were adapted to constant background light for 20 min with a quartz tungsten lamp (150 W) producing an illumination of 240 μW/cm^2^sr at the cornea. After halothane induction, animals were anesthetized with ketamine (Troy laboratories, Smithfield, Australia) and xylazine (Bayer SA, Brazil) and maintained during an experiment at 32°C with a thermoregulated bed pad. After local anesthesia with lidocaine (1%) and atropine sulfate (1%), a silver/silver chloride ring electrode was placed at the cornea and a subcutaneous platinum electrode on the skin was used as reference. A xenon lamp (LBLS-509 Sutter Instruments, Novato, CA, USA), with a monochromator (1200 lines mm^−1^ grating, ORIEL, Stratford, CT, USA) was used to produce a 500 nm narrowband (20 nm half-bandwidth) light stimulus. Optical isolation from secondary monochromator wavelength emissions was obtained by using a RG500 long-pass filter. Flash duration (10 ms under scotopic and 30 ms under photopic conditions) and flash intervals (15 or 1 s for scotopic and photopic recordings, respectively) were controlled by an electronic shutter (Uniblitz, Vincent Associates, Rochester, NY, USA) operated by custom software. The intensity range spanned between 0.065 and 65.65 photons/μm^2^ for scotopic conditions, and between 4.3 and 215 photons/μm^2^ under photopic conditions. ERG light-evoked responses were amplified with an AC/DC amplifier (A-M Systems, Model 3000, Carlsbourg, WA, USA), band-pass filtered between 1 and 100 Hz and digitalized with an A/D interface (CB-68LP, National Instruments, Austin, TX, USA). Each ERG corresponds to the average of 20–30 flashes.

### Multi-electrode recording, animal preparation, and visual stimulation

A multi-electrode array (MEA USB-256, Multichannel Systems GmbH, Germany) for *in vitro* isolated retina experiments was used to record action potential firing from a population of GCs. For MEA experiments, the animals were dark-adapted and deeply anesthetized with halothane before decapitation. Under dim red light, both eyes were enucleated and one of the retinas was diced into quarters while the other was stored in AMES medium in the dark for further experiments. For recordings, one piece of retina was mounted (GCs down) onto a dialysis membrane placed into a ring device mounted in a traveling (up/down) cylinder, which was moved to contact the electrode surface of the MEA recording array. Visual stimuli were generated by a custom software created with PsychoToolbox (Matlab) on a MiniMac Apple computer and projected onto the retina with a LED projector (PLED-W500, Viewsonic, USA) equipped with an electronic shutter (Vincent Associates, Rochester, USA) and connected to an inverted microscope (Lens 4×, Eclipse TE2000, NIKON, Japan). The image was conformed by 380 × 380 pixels, each covering 5 μm^2^. Since rodents are dichromatic (green and blue/UV cones), in our experiments with checkerboard stimuli only the B (blue) and G (green) beams of the projector were used, while the R (red) channel was used for signal synchronization. For the measurement of GC receptive fields (RF), a checkerboard stimulus with a bin size of 100 μm was used at rate of 60 fps. Optical density filters in the optical path were used to control final light intensity. A CCD camera (Pixelfly, PCO, USA) attached to the microscope was used for online visualization and calibration of the light stimuli projected onto the recording array. With the use of checkerboard as stimulus only the photopic condition was tested.

### Drug applications

The general connexin channel blocker, 18-β-glycyrrhetinic acid (β-GA, G10105, Sigma) was dissolved in ethanol (EtOH) and aliquots were diluted in PBS (0.1 M, pH 7.4). Final concentrations of EtOH were less than 0.2% in the β-GA solution. For *in vivo* ERG experiments, intravitreal injections of β-GA (10 μl of final solutions), were performed after local anesthesia (see below and Delgado et al., 2009), using a fine needle through the *ora serrata*. The volume of the rat and degu vitreous was estimated as 0.15 ml (Naarendorp and Williams, 1999) and 0.22 ± 0.02 ml, respectively, and used to calculate the final concentration of β-GA in the eye (Moller and Eysteinsson, 2003). The ERG a- and b-wave were assessed 30 min after β-GA injection from the same eye recorded previously as control. The effects of the gap junction blocker remained unaltered for several hours (data not shown). Moreover, the injection of 0.2% EtOH in PBS (10 μl) did not produce changes in the ERG amplitude (data not shown). For *in vitro* multi-electrode recording of isolated retina, β-GA (50 μM) was added to the perfusion solution 30 min before GC recording.

### Data analysis and cell classification

In ERG recordings, peak amplitudes of a- and b-wave were calculated by fitting the peak of the response to a polynomial function of fifth order using IGOR Pro Software, subtracting the averaged baseline. Results are shown as mean ± s.e.m, and statistical significance was evaluated by a paired Student's *t*-test, using Graph Pad InStat software (La Jolla, CA, USA). For MEA experiments, MC_Rack software (MultichannelSystems) served for signal visualization. After each experiment, the software Offline Sorter (Plexon, TX, USA) was used for spike sorting and Neuroexplorer (NexTechnologies, Madison, WI, USA) for further statistical analysis. Moreover, a custom software was used to calculate the spike-triggered average (STA) associated with the corresponding linear RF of each GC. As a result of the STA processing, a spatiotemporal volume characterizing the dynamics of the cellular response is obtained. In the time domain, the spatiotemporal RF is formed by 18 images corresponding to 300 ms before the spike event. The pixel with the maximal deviation from the mean was detected and a 2D Gaussian fit was adjusted in order to estimate the center of the RF. The temporal profile of the GC was obtained extracting for each frame the value of the pixel located at the position of the RF center. A STA was computed for each GC under control and β-GA conditions. Either ON or OFF cell characterization was obtained from the temporal profiles of the estimated RF. Following a procedure similar to (Chichilnisky and Kalmar, 2002; Field et al., 2007), temporal cell profiles were used for cell classification in terms of ON/OFF type. Furthermore, using principal component analysis (PCA), the temporal profiles were projected on a space with a lower dimensionality, and the classification was finally performed using k-means. Additionally, the time-to-peak parameter was measured for each temporal profile, which refers to the time of the temporal profile, previous to the occurrence of a spike, with the highest deviation being positive (GC ON) or negative (GC OFF).

## Results

### High degree of homology between retinal connexins of degu and other mammals and expression of cxs36 and 43 in degu retina

**Figure 2:**
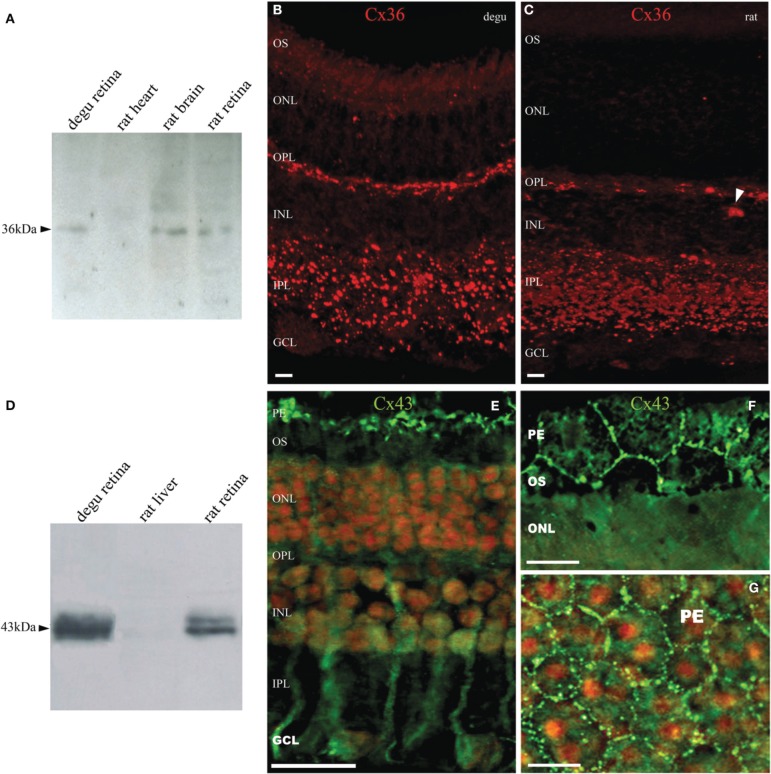
**Expression and immunolocalization of Cx36 and Cx43 in the retina of degu**. **(A)** Immunodetection of Cx36 (arrowhead) in retinal extracts of degu and rat, and brain cortex as positive control. No immunostaining was visible in heart extracts of rat. **(B,C)** Fluorescent micrograph showing Cx35/36 immunolabeling in degu and rat retina. Immunolocalization of Cx36 along the OPL and in the IPL was observed as intensely fluorescent puncta. Less frequently, Cx36 labeling was observed in the GCL, where puncta were localized to the margins of cell bodies. The arrowhead points to a blood vessel with strong non-specific labeling (**D)** Immunodetection of Cx43 in retinal extracts of degu and rat. Cx43 shows the expected electrophoretic mobility around 43 kDa. **(E)** Fluorescent micrograph of retinal Cx43 immunoreactivity in degu. The immunoreactive puncta of Cx43 were clearly visible between pigment epithelial cells (top) and possibly in glial cells present in the inner retinal layers. **(F,G)** Fluorescence micrographs of pigment epithelium layer cells showing abundant Cx43 gap junction plaques in cellular appositions. The cell nuclei were counterstained with propidium iodide staining. PE, pigment epithelial cells; OS, photoreceptor outer segment; ONL, outer nuclear layer; OPL, outer plexiform layer; INL, inner nuclear layer; IPL, inner plexiform layer; GCL, ganglion cell layer. Scale bars: 20 μm **(B,C,F,G)** and 50 μm **(E)**.

Figure 1 shows the estimated phylogenetic tree for the main connexins present in vertebrate retinas. Compared to other mammals, degu shows similarities in terms of protein sequences of the main retinal connexins. This result extends the observations of Völgyi for a large number of vertebrates (Volgyi et al., 2013a). We then studied the expression of Cx36 and Cx43, the most common neuronal and glial connexins, respectively. Cx36 immunoblotting of degu and rat retina homogenates revealed a band with an apparent molecular mass of about 36 kDa (Figure 2A). The specificity of the antibody was confirmed with rat brain cortex homogenate as positive control and heart extract from rat as negative control, due to the absence of Cx36 from this tissue. Retinal cryosections from degu and rat (Figures 2B,C) presented an intense Cx36 immunolabeling as bright dots along the OPL, where the terminals of the photoreceptors are located. Consistent with previous reports (Feigenspan et al., 2001; Frank et al., 2010) we detected weak Cx36 immunoreactivity in the OPL of rat retina, also occasionally covered by blood vessels with strong non-specific labeling (Figure 2C, white arrowhead). On the other hand, immunolocalization of Cx36 was both strong and dense within the inner plexiform layer (IPL) in both species (Figures 2B,C).

The Cx43 Western blots of degu retina homogenates show the characteristic band profile around 43 kDa (Figure 2D), which is similar to that observed in rat heart extracts, a tissue known to express high levels of this protein. No immunostaining was visible in rat liver extracts, consistent with the lack of expression of Cx43 in liver. Immunolocalization of Cx43 in degu retina showed staining in cells reminiscent of retinal Müller cells (Figure 2E) and at the borders of pigment epithelium cells (PE), revealing the prominent hexagonal array of these cells (Figures 2F,G). No immunolabeling of Cx43 was observed in other layers of degu retina. These observations are consistent with previous studies that showed a lack of Cx43 expression in retinal neurons, but a high expression in pigment epithelial cells and glial elements of several vertebrate retinas, including human (Coca-Prados et al., 1992; Janssen-Bienhold et al., 1998).

### Effect of β-GA on the B-wave of the ERG

**Figure 3:**
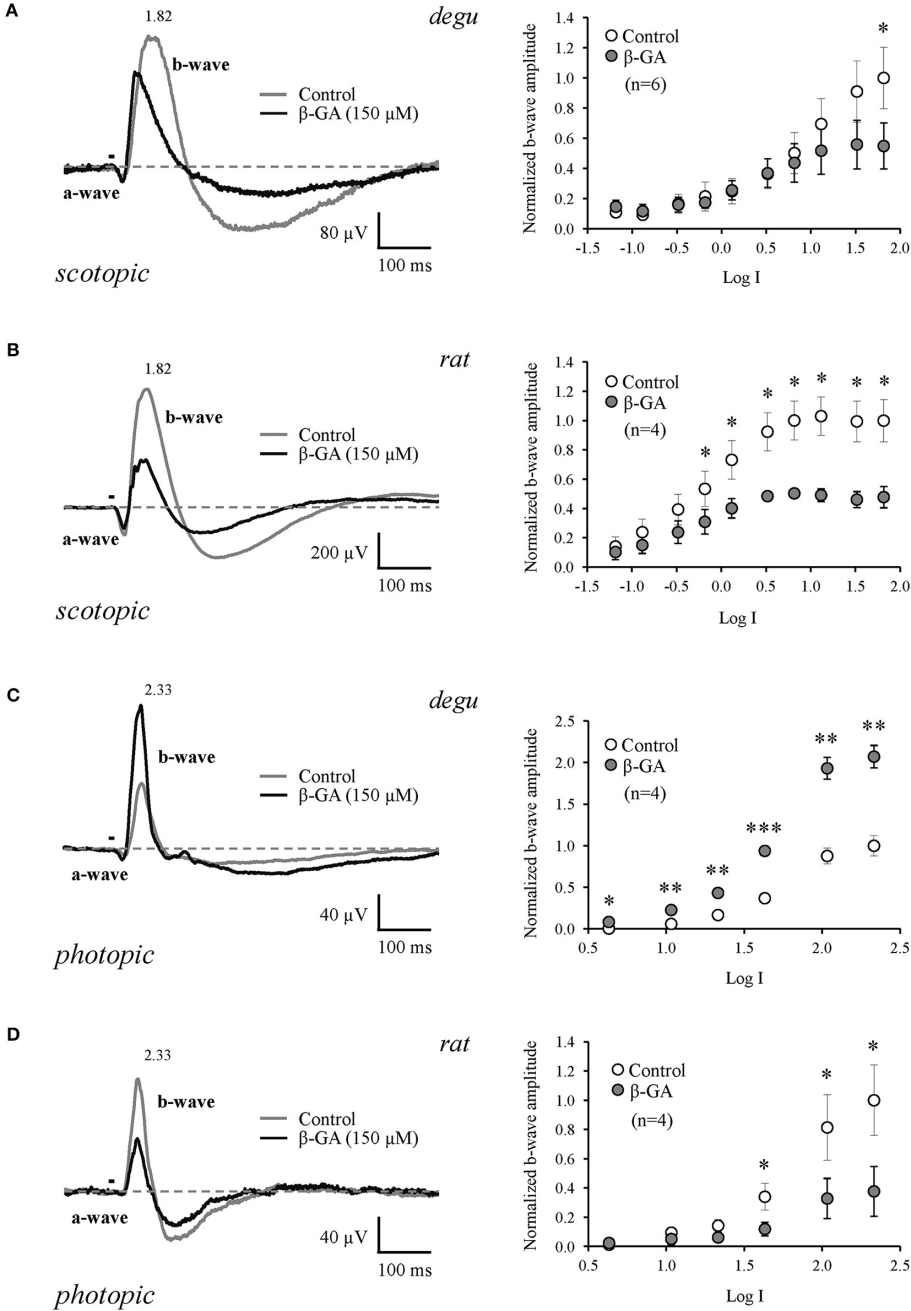
**Differential effect of connexin channel inhibition on the amplitude of the ERG b-wave in the retina of degu and rat during dark and light adapted conditions**. Left: representative ERG tracers obtained from degu **(A–C)** and rat **(B–D)** eyes under control conditions or after treatment with β-GA (150 μM), at the maximum intensity used. Bars indicate the stimulus duration (λ = 500 nm). Dotted lines indicate baseline level. Recordings were realized under dark **(A,B)** and light **(C,D)** adapted conditions. Right: intensity-response functions under dark and light conditions before and after treatments with β-GA. Asterisks represent the statistical significance with respect to control (Paired *t*-test, ^*^p < 0.05; ^**^p < 0.01; ^***^p < 0.001).

β-GA was used to investigate the contribution of gap junction channels to the generation of visual responses in degu and rat. Representative ERG response patterns are shown in Figure 3 for both species before and after injection of β-GA. Under dark-adapted conditions, β-GA produced a reduction of the ERG b-wave amplitude by 45.1 % (*n* = 6, *p* = 0.036) in degu and 52.2 % in rat (*n* = 4, *p* = 0.037) for high intensity stimuli (Figures 3A,B; Table 1). This effect was only appreciable in degu at high stimulation intensities. However, under photopic light conditions, the amplitude of the ERG b-wave increased by 107.2 % (*n* = 4, *p* = 0.006) in degu, while it was reduced by 62.3% (*n* = 4, *p* = 0.032) in rat (Figures 3C,D; Table 1). These results indicate that β-GA produced a similar global effect on both species under scotopic conditions, but opposite effects under photopic adaptation.

**Table 1:** **Summary of the effects of β-GA on the ERG parameters (mean ± s.e.m.) in degu and rat**.

**ERG-SCOTOPIC ADAPTED**						
**Log intensity**	**b-wave amplitude (μV) degu (*n* = 6)**			**b-wave amplitude (μV) rat (*n* = 4)**		
	**Control**	**β-GA**		**Control**	**β-GA**	
−0.88	9.8 ± 2.0	12.4 ± 4.7	–	136.3 ± 45.9	86.0 ± 39.1	–
0.52	38.4 ± 11.4	38.9 ± 10.0	–	526.7 ± 72.9	276.2 ± 79.9^*^	↓
1.82	105.1 ± 23.4	57.8 ± 16.0^*^	↓	569.7 ± 78.5	272.4 ± 88.85^*^	↓
**ERG-PHOTOPIC ADAPTED**						
	**b-wave amplitude (μV) degu (*n* = 4)**			**b-wave amplitude (μV) rat (*n* = 4)**		
	**Control**	**β-GA**		**Control**	**β-GA**	
1.03	2.8 ± 1.2	10.7 ± 1.5^**^	↑	6.5 ± 2.1	3.5 ± 3.2	–
1.63	17.4 ± 1.9	44.2 ± 2.2^***^	↑	23.2 ± 6.3	8.1 ± 3.9^*^	↓
2.33	47.2 ± 7.1	97.7 ± 7.8^**^	↑	68.0 ± 16.4	25.7 ± 14.2^*^	↓

Asterisks show statistically significant differences (Paired t-test;

* p < 0.05;

** p < 0.01;

*** *p < 0.001). Arrows indicate the effect tendency*.

### Ganglion cell receptive fields under control and β-GA conditions

**Figure 4:**
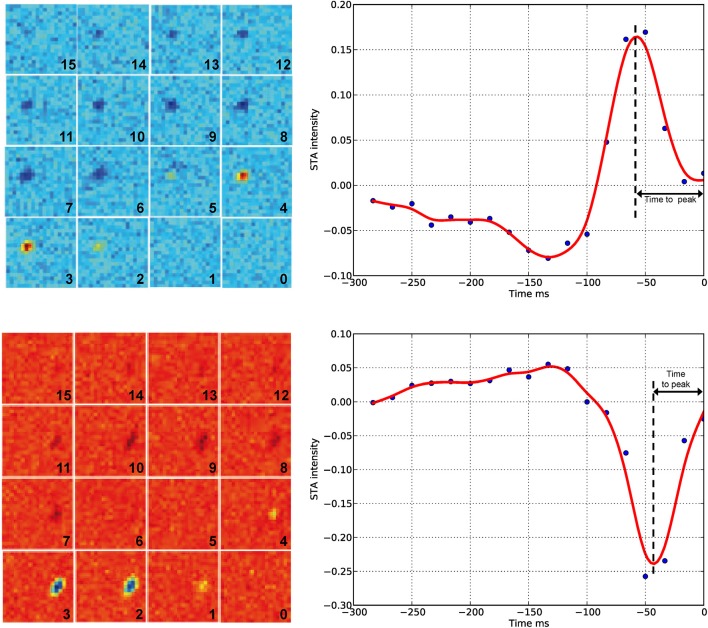
**Spike-triggered average (left) and temporal profile (right) under control conditions**. The left panel shows the result of the STA algorithm on the response of GCs to checkerboard stimuli. Each frame has a number indicating the order before the spike time (zero represents the spike occurrence time point). The complete sequence covers −250 ms before the spike time with a time resolution of 16.67 ms. Blue/red represents low/high intensities, respectively. Top, example of an ON GC (STA estimated from *n* = 2882 spikes); bottom, example of an OFF GC (STA estimated from *n* = 9263 spikes). In both cases, the light intensity before the spike occurrence is either a decrease (top) or an increase of the mean intensity of the stimuli. The right panel shows the temporal profile of the zone of the receptive field with the highest response. Time zero represents the spike occurrence. Time-to-peak represents the time between the spike occurrence and the maximum peak of the stimulus. Each blue point corresponds to a time panel figure on the left. The red line is a spline interpolation of the curve defined by the blue dots.

**Figure 5:**
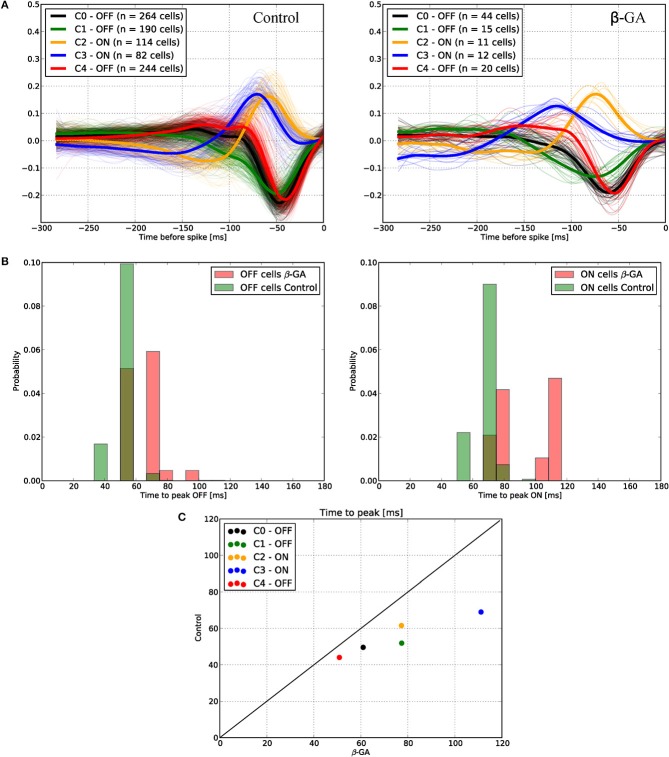
**Functional ganglion cell characterization under control and β-GA conditions**. Using STA analysis, ganglion cells were classified and clustered into five different groups according to their ON or OFF preference and response timing. **(A)** The dynamics of the response timing is represented for both control (left) and β-GA conditions (right). β-GA decreased the number of ganglion cells with a valid RF. **(B)** Distribution of time-to-peak responses under β-GA and control conditions for ON and OFF ganglion cells. Under β-GA treatment, the response latency in the temporal response profile increased compared to control. For both ON and OFF cells, the latencies observed in the β-GA condition presented a larger standard deviation compared to controls. ON cells were more affected, allowing to fit a bimodal distribution (right). **(C)** Comparison of time-to-peak vs. time-to-zero-cross for the same conditions described in **(B)**. Comparison of time-to-peak for control and β-GA treatment for the same cluster allows concluding that every cell type was affected by β-GA, in particular the ON type labeled as C3. The strait line indicates zero difference between β-GA and control.

Although we found that inhibition of connexins channels modulates the ERG b-wave, reflecting altered responses of ON BCs, the output of visual signals from the retina may not be affected in the same way by connexin inhibition. Therefore, we studied the function of connexins in degu retina at the level of GC action potentials (spikes), using a multi-electrode array for *in vitro* experiments under photopic conditions. Figure 4 shows an example of a RF (left) and its temporal profile (right) for an ON (top) and OFF (bottom) GC recorded in degu retina. Table 2 shows the distribution of ON/OFF GC types for control and β-GA conditions. Under control conditions, around 80% of GCs match type OFF, a result consistent with the literature for guinea pig and rat (Peichl, 1989; Zaghloul et al., 2003). The estimation of the linear RF allowed us to detect two main changes concerning the number of GCs and their response time with β-GA (Table 2). We observed the number of characterized GCs with a valid RF decreased under β-GA treatment for both ON (12.7%) and OFF (11.1%) type GCs. Figure 5A shows the time profile of all the GCs with a valid RF according to the criteria stated in the Materials and Methods Section for control and β-GA conditions. Fitting the time profiles with splines and using PCA and k-means, GCs clustered into five functional groups, differing in the polarity, shape and time-to-peak of their action potentials. In the presence of β-GA, the control clusters remained in place, but showed overall larger response latencies, resulting in temporal profiles with slower dynamics compared to control. The difference between the response times was parameterized using time-to-peak values (Figure 4), revealing that under both control and β-GA conditions, OFF cells are significantly faster than ON cells. Globally, the increment observed in the time-to-peak value for the two ON and OFF classes of GCs under β-GA treatment was 30.1 ms (46.42%, Welch's *t*-test *p*-value < 0.005) for ON and 13.2 ms (27.2%, Welch's *t*-test *p*-value < 0.005) for OFF type GCs (Table 2). Specifically, the effect of connexin channel blockage on the time course of the RF of different cell types is shown in Figure 5. Figure 5B shows how β-GA treatment affected the different functional GCs types in different ways. Some of the ON GCs increased their time-to-peak interval, reaching up to 117 ms compared to a mean of 64.61 ms (control). Figure 5C shows the comparison of time-to-peak latencies for the different clusters shown in Figure 5A. Evidently, a certain type of ON GC (blue cluster) is more affected by β-GA than others.

**Table 2:** **Distribution of ON and OFF retinal GCs in degu retina estimated according to their RF, using STA and checkerboard stimulation**.

**Cell type**	**Control A (*n* = 3) (%)**	**Control B (*n* = 2) (%)**	**βGA (50 μM) (*n* = 2) (%)**	**Control A time-to-peak**	**Control B time-to-peak**	**βGA (50 μM) time-to-peak**
ON	240 (20.1)	180 (20.6)	23 (23.0)	67.36 ± 12.06	64.81 ± 8.41	94.9 ± 19.3
OFF	955 (79.9)	693 (79.4)	77 (77.0)	48.60 ± 8.13	48.34 ± 7.44	61.5 ± 12.1
Total	1195 (100)	873 (100)	100 (100)			

*Control A corresponds to three different experiments (including the two in Control B) under normal conditions and represents a total of 1195 GCs. Control B and β-GA corresponds to two experiments under control (n = 873 GCs) and β-GA conditions (50 μM; n = 100 GCs). Time-to-peak corresponds to the interval between the stimulus presentation and the spike occurrence. The last two columns show the statistics of time-to-peak values (in ms, see Figure 5) for each GC type*.

Another important outcome to analyze is the number of spikes evoked by checkerboard stimulus experiments. In response to β-GA treatment, we observed a decrease in the number of evoked spikes of ON GCs from 8.2 (±7.3 SD, *n* = 115) to 4.4 (±4.2 SD, *n* = 48) spikes per second or OFF GCs from 6.2 (±5.6 SD, *n* = 265) to 3.3 (±3.1 SD, *n* = 152) spikes per second. This result suggests that in degu the increase of the photopic b-wave under β-GA treatment is not reflected by an increase in the mean GC spike rate.

## Discussion

The analysis of protein sequence relationships for the principal classes of retinal connexins of degu, guinea pig, rat and human based on the classification proposed by Cruciani and Mikalsen (2006) and discussed in Volgyi et al. (2013a) shows that most common retinal connexins are present in the degu genome and that they are closely related to other mammalian species. The phylogenetic tree in Figure 1 suggests a high degree of homology of the degu Cx36 ortholog with guinea pig, rat and human Cx36 (Volgyi et al., 2013b). Therefore, it can be expected that the anti-Cx36 rat antibody used here cross-reacts with degu Cx36. Indeed, the Cx36 antibody labeled a band with an electrophoretic mobility similar to rat Cx36 in degu retinal extracts (Figure 2A). In addition, localization of Cx36 in degu retina is mostly similar to previous findings in others rodents (Figures 2B,C). One exception is that Cx36 shows higher expression in the OPL compared to rat retina where less Cx36-positive puncta are observed, consistent with previous findings in nocturnal rodents like the rat (Figure 2C) (Frank et al., 2010). In addition, rat and degu retinas show a distributed Cx36 staining in the IPL throughout the OFF and ON sublayers, although labeling was a little more concentrated in the latter (Figure 2B), similar to previous findings in other nocturnal rodents. The punctate Cx36 staining pattern found in degu was very similar to the closely related guinea pig. In the latter, an intense staining is observed throughout the OPL, where Cx36 forms homologous gap junctions between neighboring cone-cone and rod-rod photoreceptors and forms heterologous gap junctions between cone and rod photoreceptors (Lee et al., 2003; Feigenspan et al., 2004). The higher density of Cx36 in the OPL of degu retina might be due to the presence of a significantly higher number of cones reaching a peak density of 50.000 mm^−2^ (Jacobs et al., 2003) compared to 7.000 mm^−2^ in rats (Jacobs et al., 2001). The IPL of degu retina also shows punctuate and intense staining of Cx36, particularly in the ON sublayer (Figure 2B). The localization of Cx36 immunoreactivity in the IPL is in good agreement with other mammalian retinas, where Cx36 was described in somata and dendrites of retinal amacrine AII and GCs (Feigenspan et al., 2001; Mills et al., 2001; Hidaka et al., 2002). Interestingly, in *Gallus gallus*, a diurnal bird, Cx36 is localized mainly in the OFF sublamina of the IPL (Kihara et al., 2008), suggesting that Cx36 is present in distinct retinal circuits in the mature *Gallus gallus* retina. The different distribution of Cx36 may be due to the fact that *Gallus gallus* has phylogenetically evolved an essentially rodless retina, whereas degu, although diurnal, preserves an important rod pathway consistent with the results of our study discussed below.

It is well-known that in the IPL of mammalian retina, Cx36 and Cx45 are the principal neuronal gap junction proteins, forming: “bi-homotypic” gap junction channels, with Cx45 coupling to Cx45 and Cx36 coupling to Cx36 (Li et al., 2008); or “heterotypic” gap junctions channels (Maxeiner et al., 2005; Dedek et al., 2006) between mouse AII amacrine and ON cone BC. Both Cx36 and Cx45 have an important role in visual signal transmission in the primary rod-to-cone circuit in the IPL of mammalian retina. It can be assumed that they have similar functions because it is possible to functionally replace Cx45 by Cx36 in the retina of mice (Frank et al., 2010). Furthermore, the expression and distribution of Cx43 in degu retina (Figures 2D–G) were largely similar to findings in other mammals, with staining in pigment epithelial and glial cells, Müller cells and astrocytes (Janssen-Bienhold et al., 1998; Johansson et al., 1999; Kihara et al., 2006).

We confirm previous findings showing that connexins contribute to the b-wave generated by ON BCs at the level of the OPL, where synapses between photoreceptors, bipolar and HCs are found. Under scotopic adaptation, the rat presented higher sensitivity to light compared to degu, as expected for a nocturnal animal (Table 1). However, in our full field stimulus at the highest stimulus intensity (1.82 log), the b-wave decreased in both species after β-GA application. The effect of β-GA on the ERG b-wave of rat started at lower stimulus intensities compared to degu, consistent with a robust scotopic rod system. The main scotopic rod pathway involves rods, rod ON BCs, amacrine cells and ON cone BCs; the last connection consisting of electrical synapses (Deans et al., 2002). Therefore, the blockage of Cx36 gap junctions is expected to decrease the response of the ON pathway, consistent with our findings. Along these lines of evidence, in mice, targeted disruption of Cx36 or Cx45 affects primarily the transmission of the rod pathway (b-wave) in dark-adapted retinas (Maxeiner et al., 2005). However, we cannot discard the possible contribution of Cx36 gap junctions between rods and cones in this mechanism (Deans et al., 2002), since β-GA is blocking connexin channels indiscriminately in the whole retina.

A somehow different picture emerges under photopic conditions, where the b-wave is driven essentially by the ON cone BC pathway (Deans et al., 2002), and does not involve Cx36 gap junction channels with AII cells. Under control conditions and full field stimulation, both rodent types display similar b-wave responses. However, β-GA increased the maximum amplitude of the b-wave in degu by 107%, whereas in rat it decreased the b-wave by 62% when measured at the highest light intensity (2.33 log, Table 1). In degu, β-GA increased the b-wave across nearly all stimulus intensities, whereas in rat β-GA reduced the b-wave only at the highest stimulus intensity. For rat, our results are similar to the ones observed in the Cx36^−/−^ mice (Guldenagel et al., 2001), suggesting that in both nocturnal species a similar pathways is involved, probably because their retinas are dominated by the rod pathway. Similar effects were observed in the retina of Cx45^−/−^ mice under photopic stimulation (Maxeiner et al., 2005). On the other hand, our results in degu are consistent with findings in the diurnal goldfish, where meclofenamic acid (MFA) a general gap junction blocker, also increased the photopic b-wave (Kim and Jung, 2012), suggesting that this mechanism could be present across diurnal vertebrate species.

We can only speculate about a possible explanation for the opposite effects on the photopic b-wave observed here after connexin channel blockage. Under photopic conditions, the neural circuit of the OPL involves inhibitory HCs that might play a critical role. It has been shown that HCs of type A and type B are electrically coupled, supporting an inhibitory feedback mechanism that reduces the response of photoreceptors. Interestingly, cats, rabbits and guinea pigs, the latter closely related to degu, present A and B type HCs in their retinas, while rat, mouse and gerbil have only type B HCs (Peichl and Gonzalez-Soriano, 1994; Sohl et al., 2005). Type A HCs in mammals receive mainly cone input and have a large integration area, up to 2 mm wide. In comparison, type B HCs have a smaller integration area of about 1 mm, an axon terminal that makes contact mainly with rods, and dendrites contacting cones. HCs in cats and rabbits show strong low-resistance gap junction coupling between dendrites of type A cells, whereas type B cells are less coupled (Vaney, 1994). Several types of connexins are involved in these connections. For example, in rabbits, the extensively coupled A-type HCs express Cx50 gap junctions, whereas B-type HCs axon terminals are coupled via Cx57 gap junctions (Cha et al., 2012). The absence of type A HCs from rat and their presence in degu supports a plausible anatomical and functional mechanism to explain the opposite effect of β-GA under photopic adaptation.

Another possibility is that the transition level between mesopic and photopic adaptation differs between rat and degu, and that the photopic background intensities used in the present study correspond to mesopic conditions for degu, in which rods and cones work together through an extensively connected gap junction network (Jacobs et al., 2003), while the same network would be partly disconnected in rat. In that case, β-GA would not be acting on a similar pool of active gap junctions (Bloomfield and Volgyi, 2009).

Possibly the most attractive hypothesis is that the application of β-GA could block connexin hemichannels in dendrites of HCs, preventing ephaptic transmission, which is an important feedback and gain control mechanism at photoreceptor synapses (Kamermans et al., 2001; Kamermans and Fahrenfort, 2004; Klaassen et al., 2011). The impairment of this mechanism shifts the photoreceptor membrane voltage out of the calcium channel activation range, preventing glutamate release and consequently allowing larger ON BCs responses (Klaassen et al., 2012). Similarly, a report in goldfish of an ephaptic fast inhibitory mechanism involving pannexins (Panx1) in HCs unveiled a new feedback circuit for neural light modulation that could act to increase BCs responses. ATP released by hemichannels would be a substrate for the ecto-ATPase NTPDase1 that hydrolyzes ATP to AMP, phosphate and protons, producing a buffer that keeps the synaptic cleft relatively acid, inhibiting Ca^2+^ channels of cones and reducing their glutamate release (Vroman et al., 2014). Blocking pannexin hemichannels might decrease this ephaptic inhibitory mechanism, causing alkalization of the synaptic cleft between HC and cones because of less ATP released by HCs. This would allow more release of glutamate from cones, decreasing the inhibitory surround of the BC response. However, the operation of such a mechanism still needs to be endorsed in other vertebrates including the degu model (Vroman et al., 2014).

Another degree of complexity is added by the fact that gap junctions in general interact with neuromodulatory mechanisms involving neuromodulators such as nitric oxide, dopamine, acetylcholine, regulated by the circadian rhythm and light to change retinal light sensitivity (Deans et al., 2002; Bloomfield and Volgyi, 2009). In this context, in rabbits electrical coupling of HCs decreases under scotopic and photopic conditions, but increases under mesopic adaptation (Xin and Bloomfield, 1999). In mouse, scotopic conditions were shown to increase coupling between HCs (Pandarinath et al., 2010). Furthermore, in rabbit and mouse, gap junction coupling of alpha-GCs is modulated by light. This increase in coupling is not reflected by an increase of spontaneous activity, but an increase in the correlated spike activity (Hu et al., 2010). In HCs and AII amacrine cells, gap junction coupling depends on light as well as on dopamine (DeVries and Schwartz, 1989; Bloomfield et al., 1997; Baldridge et al., 1998). The activation of dopamine receptors increases the activity of adenylate cyclase and the cytosolic concentration of cAMP, producing the activation of protein kinase A, which in turn induces the closure of gap junction channels (Piccolino et al., 1984; Lasater, 1987; DeVries and Schwartz, 1989; McMahon et al., 1989; Witkovsky and Schutte, 1991; Hampson et al., 1992; Bloomfield et al., 1997). Moreover, AII amacrine cells and ON cone BC coupling, a fundamental part of the rod pathway (Veruki and Hartveit, 2002), is modulated by nitric oxide, whose synthesis depends on light intensity (Vielma et al., 2012). This molecule activates the enzyme guanylate cyclase and increases cGMP levels producing the activation of PKG and the closure of gap junctions (Mills and Massey, 1995; Bloomfield and Volgyi, 2009).

To further understand the action of connexins and gap junctions at the level of GCs under photopic conditions, we performed multi-electrode (MEA) recordings to simultaneously measure action potential activity in hundreds of GCs. In terms of spike modulation by β-GA, these experiments revealed a decrease in the spontaneous spike activity and slower responses of ON or OFF GCs. Recently, it has been proposed that gap junctions are key players in concerted spiking in a population of GCs trough fast neural synchronization (Volgyi et al., 2013b), which may be relevant for efficient information transmission through the optic nerve (Meister and Berry, 1999; Shlens et al., 2008; Gollisch and Meister, 2010; Hu et al., 2010). In our MEA experiments, we have used a concentration of 50 μM β-GA, higher that the 25 μM of β-GA used by Völgyi (Volgyi et al., 2013b) in mouse retina. The latter work, using MEA and patch clamp experiments, reported no changes in light-evoked ionic currents of GCs, but a failure to fire synchronously in coupled GCs after β-GA application. In our experiments, β-GA application caused a decrease in the number of spontaneous spikes under dark-adapted conditions, which is to be expected. On the other hand, it is known that β-GA also blocks, in micromolar concentrations, potassium, sodium, and calcium channels. For example, in *Xenopus* cardiac cells, 30 and 50 μM of β-GA inhibited 10 and 20% of sodium currents, respectively, in a voltage-dependent manner (Du et al., 2009). To what extend this alteration of ionic channels is reflected in the neural activity of GCs observed here is not clear. Further studies are needed to fully understand the roles of connexin hemi- and gap junction channels in spike modulation at the GC level, and therefore their contribution to the retinal neural code.

### Conflict of interest statement

The authors declare that the research was conducted in the absence of any commercial or financial relationships that could be construed as a potential conflict of interest.
